# Scutellarin Enhances Antitumor Effects and Attenuates the Toxicity of Bleomycin in H22 Ascites Tumor-Bearing Mice

**DOI:** 10.3389/fphar.2018.00615

**Published:** 2018-06-14

**Authors:** Juan Nie, Hong-Mei Yang, Chao-Yue Sun, Yan-Lu Liu, Jian-Yi Zhuo, Zhen-Biao Zhang, Xiao-Ping Lai, Zi-Ren Su, Yu-Cui Li

**Affiliations:** ^1^Mathematical Engineering Academy of Chinese Medicine, Guangzhou University of Chinese Medicine, Guangzhou, China; ^2^Guangdong Province Traditional Chinese Medical Hospital, Guangzhou, China; ^3^Guangdong Provincial Key Laboratory of New Drug Development and Research of Chinese Medicine, Guangzhou University of Chinese Medicine, Guangzhou, China

**Keywords:** scutellarin, bleomycin, anti-tumor, pulmonary fibrosis, combined administration

## Abstract

Bleomycin (BLM) is a broad spectrum anti-tumor drug and inducing pulmonary fibrosis. As an anti-tumor drug without immunosuppression, it is urgent to find a drug that reduces the side effects of BLM. Scutellarin (SCU), a flavone extracted from *Erigeron breviscapus* (Vant.) Hand-Mazz, has anti-inflammatory activity and ability to inhibit tumor cell growth, migration, and invasion. However, the combined role of SCU and BLM treatment in tumor is unclear. This study aimed to investigate the possible effect and related mechanisms of BLM combined with SCU in the treatment of tumor through *in vivo* and *in vitro* experiments. *In vivo* experiments showed that BLM combined with SCU in the treatment of mice bearing H22 ascites tumor prolonged the survival time, alleviated BLM-induced pulmonary fibrosis, reduced the production of TNF-α; IL-6, and the levels of MDA and MPO. BLM combined with SCU increased the apoptotic rate of H22 ascites cells and the levels of cleaved-caspases-3 and -8. Furthermore, BLM combined with SCU increased the protein expression of p53 and gene expression of miR-29b, and decreased the expression of TGF-β1. *In vitro* experiment results showed that BLM combined with SCU inhibited the viability of H22 cells and MRC-5 cells, promoted H22 cell apoptosis, up-regulated the protein expression of p53 and down-regulated the protein expression of α-SMA and collagen-I in MRC-5 cells. These experimental results suggested that SCU could enhance the anti-tumor effect of BLM and reduce BLM-induced pulmonary fibrosis, indicating SCU as a potential adjuvant for BLM in the future.

## Introduction

Bleomycin (BLM) is a common drug used for the treatment of several types of neoplasms, such as skin carcinoma, ovarian cancer, esophageal carcinomas, and so on (Della Latta et al., 2015). BLM is an efficacious anti-cancer chemotherapeutic agent, but still has side effects that cannot be ignored. BLM induces pulmonary fibrosis, which is a chronic and aggressive lung disorder with only few successful treatments available (Abuelezz et al., 2016). Today, most of the chemotherapeutic drugs have side effects; including bone marrow suppression and immunosuppression. However, BLM, as an anti-tumor drug, caused no damage to the immune system and bone marrow (Sugiyama and Kumagai, 2002). Studies have reported that BLM in combination with some other drugs reduced the side effects of BLM-induced pulmonary fibrosis (Burgy et al., 2016). Therefore, it is necessary to investigate the adjuvants that enhance the antitumor effects and reduce the side effects of BLM-induced pulmonary fibrosis.

Scutellarin (SCU), a flavone extracted from *Erigeron breviscapus* (Vant.) Hand-Mazz, is clinically used to treat patients with ischemic heart diseases and paralysis caused by cerebrovascular diseases in China. Recent studies have reported that SCU can be used for the treatment of hypertension, Alzheimer’s disease, Parkinson’s disease, and neurodegenerative diseases and also for the prevention of cerebral thrombosis, cerebral hemorrhage, and ischemic injury through animal models (Lin et al., 2007; Pan et al., 2010; Niu et al., 2015; Shi et al., 2015; Dong et al., 2016; Wang et al., 2016). Few experiments verified SCU in protection against lipopolysaccharide-induced acute lung injury in mice (Tan et al., 2010). In addition, SCU inhibits cancer by suppressing the growth and invasion of cancer cells and inducing cancer cell apoptosis (Li et al., 2013; Han et al., 2017). However, few reports combined the use of SCU and anticancer drugs. Our study aimed to determine whether SCU can enhance anti-tumor effect and reduce the side effects when combined with BLM in the treatment of ascites tumor.

In this study, we sought to investigate the synergistic and attenuated effects and possible underlying mechanism of SCU on BLM both *in vivo* and *in vitro*. For *in vivo* experiments, we used the typical H22 tumor-bearing mice model to investigate our opinion. For *in vitro* experiments, we used MRC-5 and H22 cell lines to probe whether SCU can strengthen the efficacy of BLM treatment and reduce BLM-induced side effects of pulmonary fibrosis.

## Materials and Methods

### Experimental Drugs and Instruments

Scutellarin was purchased from Shanghai Rong Wo Pharmaceutical Technology Co. (China). BLM hydrochloride was obtained from Haizheng Pharmaceuticals (Zhejiang, China). Interleukin-6 (IL-6) and tumor necrosis factor-α (TNF-α) ELISA kits were obtained from eBioscience (San Diego, CA, United States). Myeloperoxidase (MPO) and malondialdehyde (MDA) Colorimetric Activity Assay Kits were obtained from Jiancheng Institution of Biotechnology (Nanjing, China). Annexin V-fluorescein isothiocyanate (FITC) apoptosis kit was purchased from KeyGen Biotech (Nanjing, China). TRIzol reagent was obtained from Invitrogen Life Technologies (Shanghai, China). All other chemicals and reagents used in the study were of analytical grade.

### Cell Culture

Mouse liver cancer H22 cells (H22) and Human Embryonic Lung fibroblasts MRC-5 cells (MRC-5) were obtained from American Type Culture Collection (Rockville, MD, United States). H22 cells were cultured in RPMI 1640 medium (Gibco-BRL Co., Ltd., United States) with 10% fetal bovine serum (FBS, Gibco-BRL Co., Ltd., United States) and 1% penicillin–streptomycin (Hyclone, Co., Ltd., Logan, UT, United States). MRC-5 cells were incubated in DMEM medium (Gibco-BRL Co., Ltd., United States) containing 10% FBS and 1% penicillin–streptomycin. All cells were incubated in a humidified atmosphere of 5% CO_2_ at 37°C.

### Animal Experiments

SPF male Kun Ming (KM) mice, weighting 18–22 g, were provided by the Experimental Animal Center, Institute of Guangzhou University of Chinese Medicine (Certificate number SCXK2008-0020; Ethical permission date was September 21, 2015, Guangdong Province, China). The animals were housed in a 12-h light/dark cycle under a constant temperature of 24°C and relative humidity of 65 ± 15% and fed with standard diet and tap water. The animal experiments were conducted according to the guidelines established by the NIH Guide for the Care and Use of Laboratory Animals. All experimental protocols were followed Animal Care and Use Committee at Guangzhou University of Chinese Medicine.

### H22-Bearing Mice and Treatment

H22 cells (2 × 10^6^ cells/ml) were inoculated into the abdomen of male KM mice and the ascites cells were passaged three times in the mice, after 1 week, the ascites was collected and diluted with normal saline; the cell concentration was adjusted to 2 × 10^6^ cells/ml and injected into each mouse. After 5 days, 90 mice were randomly divided into nine groups of 10 mice. The control group: intraperitoneal (ip) injection of normal saline, model group: normal saline, ip; BLM alone group: BLM (7.5 mg/kg, ip., the pre test results were shown in the Supplementary Figures S1, S3), SCU-L, M, H doses alone group: intragastric (ig) administration of SCU (30 mg/kg, 60 mg/kg, 90 mg/kg), BLM combined with SCU-L, M, H doses group: intraperitoneal injection of BLM (7.5 mg/kg) combined with intragastric administration of SCU (L: 30 mg/kg, M: 60 mg/kg, H: 90 mg/kg). After 24 h, the control and model group were intraperitoneally injected normal saline, and BLM alone group with BLM. The SCU alone group were, respectively, gavaged SCU-L, M, H solvent; the BLM combined with SCU groups were, respectively, intraperitoneally injected BLM and gavaged SCU-L, M, H solvent once per day. Each group of mice were given free access of diet and the survival time of each mouse was recorded.

The another 50 mice were randomly divided into five groups with 10 mice in each group: the control group and model group (normal saline, ip), BLM alone group (7.5 mg/kg, ip), SCU-M alone group (60 mg/kg, ig), and BLM (7.5 mg/kg, ip) combined with SCU-M (60 mg/kg, ig) group. Each group of mice was administered once a day as describe above for seven consecutive days. The weight of the mice and the abdominal diameter were measured every day. All mice were sacrificed on day 8 to collect ascites and lung tissues for the subsequent tests. The volume of ascites was measured at the time of collection. The lung tissues were quickly removed and washed with cold normal saline. A portion of the ascites was solubilized in the TRIzol regent for the extraction of total RNA, and the other portion was used for Western blotting analysis. The lung tissue stored in a -80°C refrigerator until further analysis.

### H22 Cell Viability Test

[3-(4,5-dime-thylthiazol-zyl)-5-(3-carboxymethoxyphenyl)-2-(4-sulfophenyl)-2H-tetrazoli-uzolium, inner salt] (MTS) (Sigma-Aldrich Co., Ltd., United States) assay was used to measure the inhibition rate of SCU, BLM, and SCU combined with BLM in H22 cells. H22 cells (1 × 10^5^ cells/ml) were plated in 96-well plates with 200 μl in each well. RPMI 1640 medium was added into the blank group without cells, the control group with only cells, and the experimental group containing BLM (12.5 μM, 25 μM, 50 μM, 100 μM, 200 μM), SCU (10 μM, 20 μM, 40 μM, 80 μM, 100 μM, 120 μM) and BLM combined with SCU [BLM (5 μM) + SCU (5 μM), BLM (10 μM) + SCU (10 μM), BLM (20 μM) + SCU (20 μM), BLM (30 μM) + SCU (30 μM), BLM (40 μM) + SCU (40 μM)]. After culturing for 24 and 48 h, 20 μl of MTS was added to each well. The cells then incubated continuously for 4 h, and the optical density (OD) was measured with micro-plate reader at a wavelength of 490 nm.

### Combined Effect Evaluation

The BLM and SCU interaction in cell culture was assessed at a fixed concentration ratio of 1:1, using the combination index (CI) method published by Chou and Talalay (Chou, 2006, 2010). Cell viability assays were used MTS assay. CI values were calculated with CompuSyn 2.0 software. The values for CI more than 1 (CI > 1), equal to 1 (CI = 1), and less than 1 (CI < 1) indicate antagonistic, additive and synergistic effects, respectively.

### MRC-5 Cell Viability Test

MRC-5 cells (2 × 10^4^ cells/ml) were seeded into 96-well plates at 200 μl per well. One day after seeding, the cells were dealt with fresh DMEM medium. In BLM and SCU alone group, after the cell density reaches 60–70% confluence, the DMEM medium was removed and 200 μl fresh DMEM containing BLM (12.5 μM, 25 μM, 50 μM, 100 μM, 200 μM), and SCU (10 μM, 20 μM, 40 μM, 80 μM, 100 μM, 120 μM) were added to the wells. The culture was continuously incubated for 24 h (48 h), and then 20 μl MTS was added to each well. The OD was measured at a wavelength of 490 nm after 4 h in a micro-plate reader. In the BLM combined with SCU group, after the cell density reaches 60–70% confluence, the old culture medium was removed and 200 μl fresh DMEM containing BLM (30 μM) was added to the wells. After culturing for another 24 h, the DMEM medium was removed and the cells were treated with new medium containing SCU (6.25 μM, 12.5 μM, 25 μM). After incubation for 24 h (48 h), 20 μl MTS was added to each well. After 4 h incubation, the OD value was measured at a wavelength of 490 nm in a micro-plate reader.

### Flow Cytometry Analysis

Apoptosis was evaluated by staining the cells with Annexin V-FITC apoptosis. Ascites was centrifuged (1,000 rpm, 3 min) and then washed twice with cold PBS. After that, the cell concentration was adjusted to 1 × 10^6^ cells/ml. Then 400 μl 1× Annexin V staining buffer and 5 μl Annexin V-FITC was added to the cell suspension, incubated for 15 min at 4°C in the dark and added 10 μl propidium iodide (PI) for staining. After 5 min incubation at 4°C in the dark, the samples were then immediately analyzed by flow cytometry (BD Biosciences, San Jose, CA, United States).

H22 cells (2 × 10^5^ cells/ml) were seeded into 6-well plates with 2 ml in each well. RPMI 1640 medium containing BLM(30 μM), SCU (25 μM), BLM (30 μM) combined with SCU (25 μM) were immediately added to the culture. After 48 h, suspended cells were collected and washed twice with cold PBS. PBS was removed, the cell concentration was adjusted to 1 × 10^6^ cells/ml and 400 μl of 1× Annexin V staining buffer and 5 μl Annexin V-FITC were added to the above cell suspension and incubated for 15 min at 4°C in the dark. Then 10 μl PI staining solution was added to the cell suspension and incubated for 5 min at 4°C in the dark. Cells were analyzed using flow cytometry.

### Cleaved-Caspase-3 and Cleaved-Caspase-8 Activities Assay

H22 ascites’ cleaved-caspases-3 and -8 activities were evaluated through caspase activity assay kit obtained from KeyGen Biotech (Nanjing, China) according to the manufacturer’s instructions. The related caspase activities can be quantified by spectrophotometric detection of free pNA (λ = 405 nm) after cleavage from the peptide substrate (amino acid sequence DEVD-pNA for caspase-3 and sequence IETD-pNA for caspase-8), using a spectrophotometer.

### Histopathological Examination

The lung tissues were fixed in 10% buffered formalin, and embedded in paraffin. Paraffin sections (4 μm thick) were stained with hematoxylin and eosin (H&E) and Masson’s Trichrome and examined under light microscope to determine lung tissue inflammation or collagen deposition. The lung histological changes were evaluated according to the published methods (Yin et al., 2008). (a) Interstitial inflammation (score: 0–4), (b) inflammatory cell infiltration (score: 0–4), (c) congestion (score: 0–4), and (d) edema (score: 0–4) (Yin et al., 2008). The final lung injury score was composed of the sum of individual scores of each category.

### Determination the Expression of TNF-α and IL-6

Saline was added to the lung tissues (0.3 g) a ratio of 1:9. Hereafter, samples were homogenized (1,000 rpm, 30 s) and centrifuged (3,000 ×*g*, 4°C, 10 min). The expression levels of TNF-α and IL-6 were detected using TNF-α and IL-6 ELISA kit according to the manufacturer’s instructions.

### Measurement of MDA and MPO Levels

The MDA and MPO levels were measured by MDA and MPO Activity Assay Kits. MDA was measured by the TBA method and MPO was determined by colorimetric (Shi et al., 2010). The lung tissues (0.2 g) were operated according to the manufacturer’s instructions: homogenized (1,000 rpm, 30 s) with cold normal saline (4°C), and then the MDA and MPO levels were measured by the Assay Kits according to the manufacturer’s instructions.

### Immunofluorescence Analysis

The MRC-5 cells were seeded into 6-well plates with slides of 2 ml in each well. After the cell density reaches to 70–80%, the cells were treated with BLM (30 μM) for 24 h and then treated with SCU (25 μM). After 24 h, the cells were washed three times with cold PBS, fixed with 4% paraformaldehyde for 15 min, washed with PBS for three times, 3 min each, and then permeabilized in 0.5% Triton X-100 (Dingguo prosperous biotechnology, Beijing, China) at room temperature for 20 min. After that, the slides were washed with PBS for three times, 3 min each, and then closed with normal goat serum at room temperature. After 30 min, the cells were incubated overnight with anti-α-SMA (1:100, Sigma-Aldrich, St. Louis, MO, United States) at 4°C. Next day after washing with PBS three times, the cells were incubated for 1 h at 20–37°C with DyLight 594 AffiniPure goat Anti-Mouse IgG (H+L) (1:200, AmyJet Scientific Inc., Wuhan, China). Then the cells were washed three times with PBS, and incubated with 4′-6-diamidino-2-phenylindole (DAPI) (AmyJet Scientific Inc., Wuhan, China). Cell fluorescence was then observed under a fluorescence microscope.

### Western Blot Analysis

H22 cells were plated into 6-well plates with 2 ml per well. Control group and the experiment group were treated with 1640 medium and 1640 medium containing BLM (30 μM), SCU (25 μM), or BLM (30 μM) combined with SCU (25 μM). After 24 h, the cells were washed for three times by cold PBS and were collected. The MRC-5 cells were seeded into 6-well plate with slides of 2 ml in each well. After the cells reached 70–80% confluence, the cells were treated with BLM (30 μM) for 24 h and then treated with SCU (25 μM). The culture was then incubated for 24 h again. The cells were washed three times with cold PBS and then were collected.

The total protein was collected. The collected ascites cells, H22 cells, MRC-5 cells and the lung tissues were washed three times with cold PBS, centrifuged (1,000 ×*g*, 5 min, 4°C), and then the supernatant was collected. 200 μl RIPA buffer was added, and then centrifuged at 12,000 ×*g* for 5 min at 4°C. The above steps are operated on ice. The cell plasma and nuclear proteins were collected by cell plasma protein and nucleus protein kits. The protein concentration was measured by BCA method. Equal amounts of proteins were separated using 10% SDS polyacrylamide gel electrophoresis and then transferred onto polyvinylidene fluoride (PVDF) membranes. After blocking for 1 h in PBS containing 5% non-fat dry milk and incubating overnight at 4°C with primary antibodies specific for TGF-β1 (1:500, Santa Cruz Biotechnology, Inc., Shanghai, China), p53 (1:500, Santa Cruz Biotechnology, Inc., Shanghai, China), α-smooth muscle actin (α-SMA) (1:500, Daiichi Fine Chemical Co., Ltd., Shanghai, China) and type I collagen (collagen-I) (1:500, Daiichi Fine Chemical Co., Ltd., Shanghai, China), and Actin (1:1000, Abcam, England). The membranes were washed three times with TBST containing a secondary antibody and then incubated for 2 h at room temperature. The super signal west Pico chemiluminescent substrate was used to detect the protein bands.

### Real-Time Quantitative PCR

Total RNA of the H22 ascites fluid and lung tissues were extracted using TRIzol reagent. Then 1.5 μg total RNA was reverse transcribed by cDNA reverse transcription according to the kit instruction (Beijing Ming Yang Kehua Biological Technology Co., Ltd., China). The reaction was carried out using Applied Bio Systems Step-one Fast Real-Time PCR system under the following conditions: 50°C for 2 min, 95°C for 10 min, followed by 40 cycles for 15 s (95°C) and 1 min (60°C), then annealed at 60°C for 60 s and extended for 40 s at 72°C with a final extension for 7 min at 72°C. Real-time qPCR products were analyzed following the amplification of the products and their respective primers were presented in **Table 1**. β-actin was selected as an internal control and sample variation was corrected by subtracting the β-actin. The relative expression of miR-29b was calculated using the 2^-ΔΔCt^ method. Fold change = 2^-ΔΔCt^, ΔΔCt = (Ct _Sample_ - Ct _β-actin_) - (Ct _Control_ - Ct _β-actin_).

**Table 1:** Primers sequences used for quantitative PCR.

Gene name		Primer (5′-3′)
β-actin	Forward	GTCCCTCACCTCCCAAAAG
	Reverse	GCT GCC TCA ACA CCT CAA CCC
miR-29b	Forward	CTCAACTGGTGTCGTGGAGTCGGCAATTCAGTTGAGT
		CTAAACC
	Reverse	ACACTCCAGCTGGGGCTGGTTTCATATGGTGG


### Statistical Analysis

All data were expressed as mean ± standard deviation (SD). Data analyses were performed using SPSS 23.0 software. Data analyses of different groups were performed using Student’s *t*-test, one-way ANOVA and Least Significant Difference (LSD), followed by Tukey’s *post hoc* test. Log-rank (Mantel-Cox) test was applied to analyze the comparison of survival curves. *P-*value < 0.05 was considered as statistically significant. Graph were drawn in the GraphPad Prism software (version 6).

## Results

### Anti-tumor Effects of SCU, BLM, and Their Combination in Ascites Tumor-Bearing Mice

**FIGURE 1:**
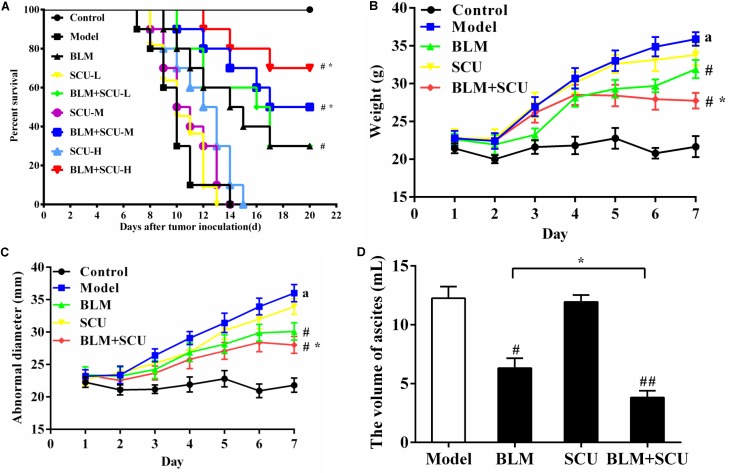
Effects of SCU, BLM, and their combination on tumor-bearing mice. **(A)** The survival curves of mice. The survival rate was followed up to 22 days after inoculation. **(B)** The weight of mouse. **(C)** The abnormal diameter of mouse. **(D)** The ascites volume of mouse. Each point represents the mean ± SD. (*n* = 8). ^#^*p* < 0.05 compared with model group; ^##^*p* < 0.01 compared with model group; ^∗^*p* < 0.05 compared with BLM group; *a* < 0.05 compared with control group.

To study the anti-tumor effects of SCU, BLM and their combination in ascites tumor-bearing mice, we observed the survival time of the mice. As shown in **Figure 1A**, compared with model group, the anti-tumor effect of SCU (L: 30 mg/kg, M: 60 mg/kg, H: 90 mg/kg) alone group showed no obvious survival in mice (*p* > 0.05), but vice versa are the results in BLM alone group (*p* < 0.05). Moreover, mice treated with BLM (7.5 mg/kg) + SCU-M, H (M: 60 mg/kg, H: 90 mg/kg) significantly prolonged the survival time when compared with BLM alone group (*p* < 0.05). These results indicated that SCU-M, H (M: 60 mg/kg, H: 90 mg/kg) could improve the anti-tumor effects of BLM. Hence, SCU at a dose of 60 mg/kg was used in the following experiments.

**Figures 1B–D** also displayed the synergistic effects of BLM combined with SCU (60 mg/kg) in the treatment of tumors. Compared with control group, the weight gain (**Figure 1B**), abnormal diameter (**Figure 1C**) and volume of ascites degree of change (**Figure 1D**) were seen. While compared with the model group, the above three indicators in the BLM alone group and BLM combined with SCU (60 mg/kg) group were significantly decreased (*p* < 0.05). In addition, the mice in the treatment group showed a more vigorous state, while the mice in the BLM combined with SCU (60 mg/kg) group showed better response. However, when compared with the model group, weight, volume of ascites and abdominal diameter of SCU (60 mg/kg) alone group showed no difference. These results suggested that SCU (60 mg/kg) could improve the anti-tumor effect of BLM when combined, but SCU (60 mg/kg) alone perhaps had little anti-tumor effect.

### Effects of BLM, SCU, and Combination of Both on the Inhibition Rate of H22 Cells

**FIGURE 2:**
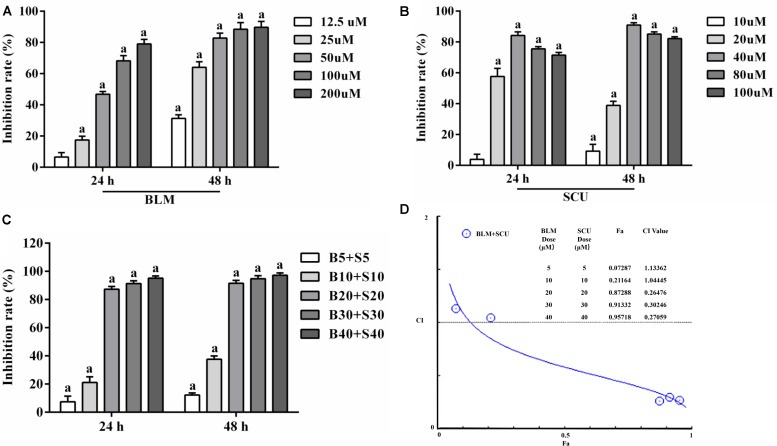
Effects of BLM, SCU, and their combination on the H22 cells. **(A)** The inhibitory rate of BLM on H22 cells after 24 and 48 h. **(B)** The inhibitory rate of SCU on H22 cells after 24 and 48 h. **(C)** The inhibitory rate of BLM combined with SCU on H22 cells after 24 and 48 h. The inhibitory rate = 1 – (OD _Experimental_ – OD _Blank_/OD _Control_ – OD _Blank_ × 100%). **(D)** The combination index (CI) values were determined. CI vs. fraction affected (Fa) plots were analyzed with CompuSyn 2.0 software. Data are expressed as the mean ± SD. (*n* = 5). *a* < 0.05 compared with control group.

To verify the synergistic effects of SCU on BLM *in vitro*, we measured the inhibition rate of SCU combined with BLM in H22 cells. **Figure 2** showed the effect of BLM, SCU and the combination of the two in H22 cells. Compared with the control group, the inhibition rate of H22 cells treated with BLM (**Figure 2A**) was significantly increased with the increased concentration. In the combination group (**Figure 2C**), the rate of inhibition was significantly higher than that of the BLM and SCU alone. Data in the graph showed that BLM and SCU alone could inhibit the growth of H22 tumor cells *in vitro*, and inhibited when combined. In addition, the combination of BLM and SCU [BLM (20 μM) + SCU (20 μM), BLM (30 μM) + SCU (30 μM), BLM (40 μM) + SCU (40 μM)] showed a synergistic effect with CI < 1 (**Figure 2D**). The pre test results are shown in the Supplementary Figure S2.

### Effects of BLM Combined With SCU in the Induction of H22 Cell Apoptosis

**FIGURE 3:**
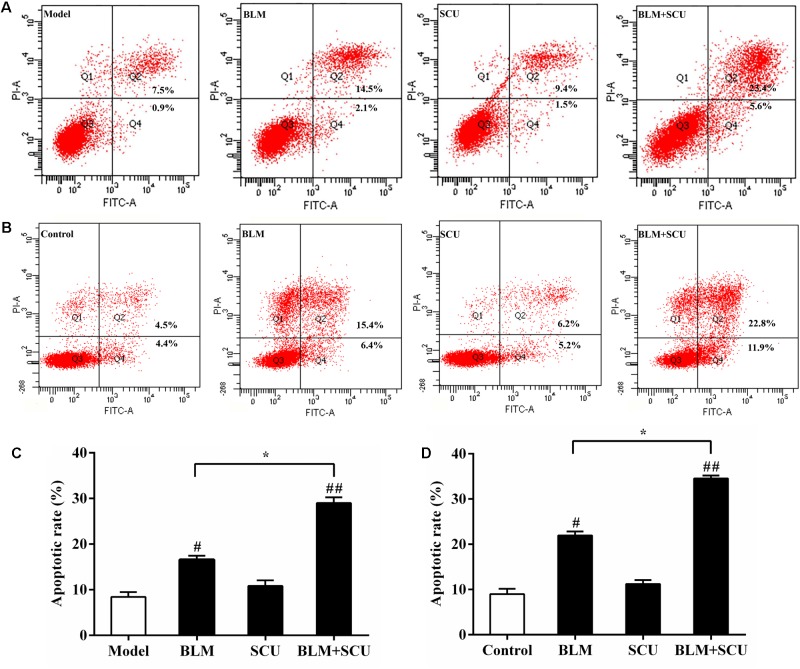
Effects of BLM, SCU, and their combination induce apoptosis of H22 ascites cells and the H22 cells cultured *in vitro*. The apoptosis rate of **(A)** H22 ascites cell and **(B)** the H22 cells cultured *in vitro* were analyzed by Flow cytometry. **(C)** H22 ascites cell and **(D)** *in vitro* H22 cell apoptotic rates were calculated. The apoptotic cells in the Q2 region are late apoptotic cells stained with FITC+/PI+ and the apoptotic cells in the Q4 region are early apoptotic cells stained with FITC+/PI–. The total apoptotic rate of cells was the sum of the apoptotic rates of Q2 and Q4. Data are expressed as the mean ± SD. (*n* = 4). ^#^*p* < 0.05 compared with model group; ^##^*p* < 0.01 compared with model group; ^∗^*p* < 0.05 compared with BLM group.

For *in vivo* and *in vitro* experiments, flow cytometer was used to analyze the apoptotic rate of H22 ascites cells in each group. **Figures 3A,C** showed H22 cell apoptotic rate in ascites and **Figures 3B,D** showed H22 cell apoptosis in *in vitro* cultured H22 cells. Results showed that in the BLM alone group and BLM+SCU combined group, the apoptotic rate of H22 ascites cells was increased when compared with model group or control group (all *p* < 0.05). The apoptotic rate of H22 cells was increased in BLM (7.5 mg/kg) + SCU (60 mg/kg) combined group when compared with BLM alone group (*p* < 0.05). All these data suggested that SCU combined with BLM could induce apoptosis of H22 cells, meaning that SCU can enhance the anti-tumor effects of BLM.

### Effect of BLM Combined With SCU on Cleaved-Caspases-3 and -8 Activities in Ascites

**FIGURE 4:**
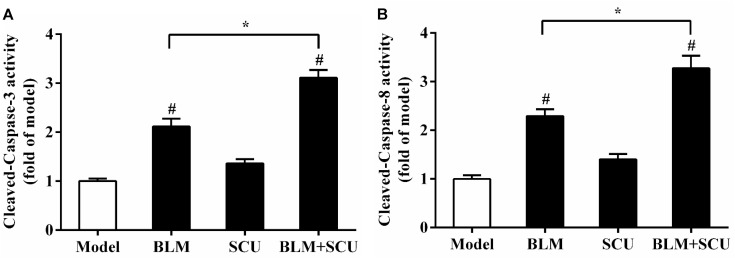
SCU enhanced the anti-tumor effect of BLM by modulating the activities of cleaved-caspase-3 and cleaved-caspase-8 in tumor-bearing mice. The activities of cleaved-caspase-3 **(A)** and cleaved-caspase-8 **(B)** were measured. Data are presented as the mean ± SD of the changes compared to the model group. (*n* = 8). ^#^*p* < 0.05 compared with model group; ^∗^*p* < 0.05 compared with BLM group.

The caspase family has long been considered to be closely related to apoptosis, especially cleaved-caspase-3 and -8. So, to demonstrate that SCU can enhance the anti-tumor effects of BLM by promoting tumor cell apoptosis, we measured cleaved-caspase-3 and cleaved-caspase-8 activities. **Figure 4** showed that the activities of cleaved-caspase-3 (**Figure 4A**) and cleaved-caspase-8 (**Figure 4B**) were enhanced in BLM alone group (*p* < 0.05) and SCU (60 mg/kg) alone group when compared with model group. The activities of cleaved-caspase-3 and cleaved-caspase-8 in SCU (60 mg/kg) and BLM (7.5 mg/kg) combined group were more significantly up-regulated when compared with BLM alone group (*p* < 0.05). Experimental results indicated that when SCU combined with BLM, the effect of BLM on the activities of cleaved-caspase-3 and cleaved-caspase-8 was enhanced. So, the combination of SCU and BLM promoted the apoptosis of tumor cells.

### SCU Attenuated Lung Fibrosis Induced by BLM

**FIGURE 5:**
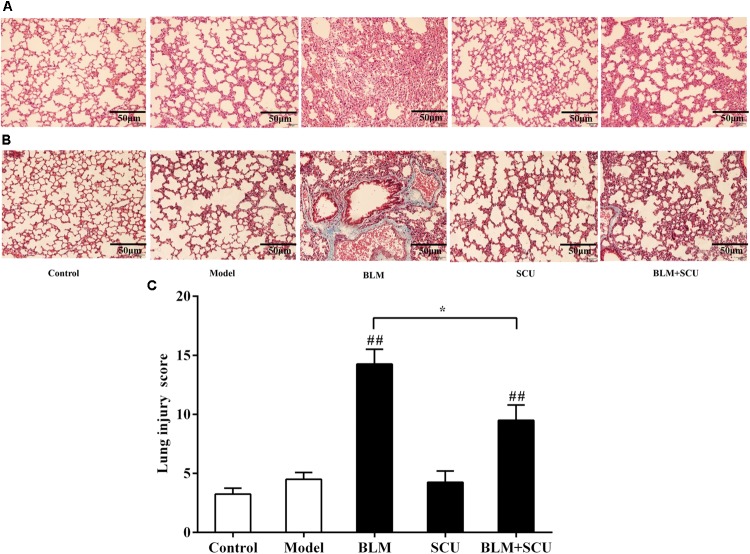
Effects of SCU attenuated BLM-induced lung fibrosis. Lung tissue sections were stained with hematoxylin–eosin (HE) staining **(A)** for pathological observation (×200), with Masson **(B)** for collagen deposition (×200). **(C)** The lung injury scores; the slides were histopathological evaluated using a semi quantitative scoring method. (a) Interstitial inflammation (score: 0–4), (b) inflammatory cell infiltration (score: 0–4), (c) congestion (score: 0–4), and (d) edema (score: 0–4). The total lung injury score was calculated by adding up the individual scores of each category. Scale bar indicates 50 μm. Data are shown as the mean ± SD. (*n* = 4). ^##^*p* < 0.01 compared with model group; ^∗^*p* < 0.05 compared with BLM group.

**Figure 5** revealed no appearance of inflammation or collagen deposition in the lung tissue of the control group. However, when compared with control group, the lung tissue of the model group and SCU (60 mg/kg) alone group did not differ much from the control groups, but the BLM (7.5 mg/kg) alone group showed inflammatory cell infiltration and vascular congestion in H&E staining. And the lung injury scores of all samples were shown in **Table 2**. Masson’s staining demonstrated collagen deposition in the lung interstitium and around bronchioles. In contrast, compared with BLM alone group, the inflammation and collagen deposition of BLM (7.5 mg/kg) combined with SCU (60 mg/kg) group were reduced. All these results suggested that BLM combined with SCU for tumor treatment; significantly alleviated pulmonary fibrosis.

**Table 2:** The lung injury scores of all samples (*n* = 4).

	a	b	c	d	Total score
	1	1	1	1	4
	0	0	2	1	3
Control	1	0	2	0	3
	1	1	0	1	3
					
	1	2	0	2	5
Model	1	1	1	1	4
	1	1	2	1	5
	1	2	0	1	4
					
	4	4	2	4	14
BLM	3	4	3	4	14
	4	4	2	3	13
	4	4	4	4	16
					
	1	1	1	1	4
SCU	1	1	0	1	3
	1	2	1	1	5
	1	1	2	1	5
					
	3	2	2	3	10
BLM + SCU	3	2	2	2	9
	3	3	2	3	11
	2	3	1	2	8

*^*a*^Interstitial inflammation (score: 0–4); ^*b*^inflammatory cell infiltration (score: 0–4); ^*c*^congestion (score: 0–4); ^*d*^edema (score: 0–4).*

### Effects of BLM, SCU, and Combination of Both on the Cell Viability of MRC-5 Cell

**FIGURE 6:**
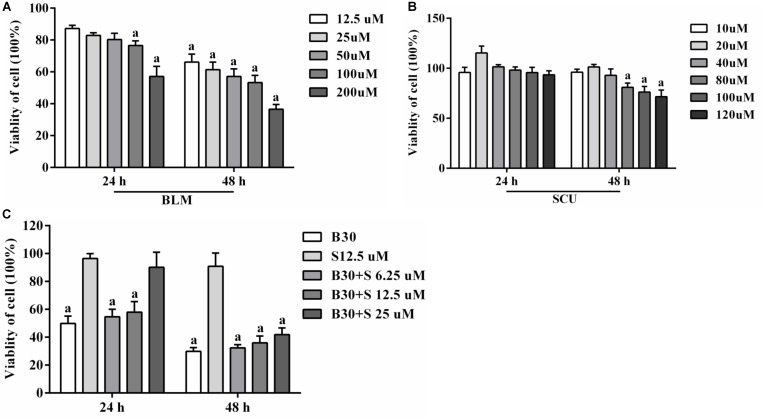
Effects of BLM, SCU, and their combination on the viability of MRC-5 cells. **(A)** The viability of MRC-5 cells after 24 and 48 h treatment of BLM alone. **(B)** The viability of MRC-5 cells after 24 and 48 h treatment of SCU alone. **(C)** The viability of MRC-5 cells after 24 and 48 h treatment of BLM combined with SCU. The data of the control group were pegged as 0.0%, whereas other data were calculated relative to it. Data are shown as the mean ± SD. (*n* = 5). *a* < 0.05 compared with control group.

MTS assay was used to measure the effect of SCU and BLM on MRC-5 cell viability. In **Figure 6**, we observed that BLM (**Figure 6A**) can reduce the cell viability of MRC-5 cell when compared with control group (*p* < 0.05). However, when the MRC-5 cells were treated with SCU (**Figure 6B**, 20 μM), the cell viability remained the largest. Then, the cell viability was gradually decreased. These data showed that SCU could inhibit the viability of MRC-5 cells at a range of concentrations *in vitro*. The pre test results are shown in the Supplementary Figure S2.

### Production of Cytokines Induced by BLM in the Lung Tissues

**FIGURE 7:**
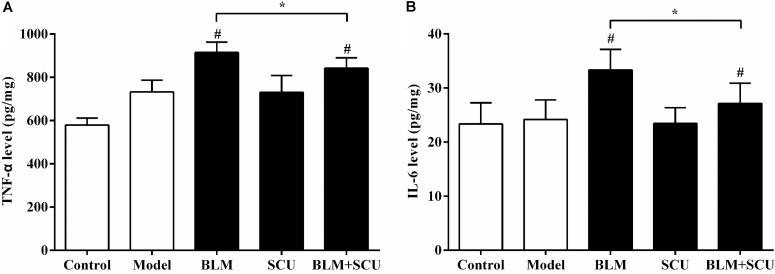
Effects of SCU, BLM, and their combination on cytokine production in lung tissues of H22 tumor-bearing mice. **(A)** The levels of TNF-α in lung tissues. **(B)** The levels of IL-6 in lung tissues. The data are shown as the mean ± SD. (*n* = 8). ^#^*p* < 0.05 compared with model group; ^∗^*p* < 0.05 compared with BLM group.

The extent of inflammation could generally be reflected by the level of cytokines produced. So, we measured the levels of TNF-α and IL-6 to evaluate the extent of inflammation in the lung tissues. As shown in **Figure 7**, when compared with the model group, the levels of TNF-α (**Figure 7A**) and IL-6 (**Figure 7B**) in the BLM alone group were increased significantly (*p* < 0.05). In contrast, the levels of TNF-α and IL-6 in the SCU (60 mg/kg) and BLM (7.5 mg/kg) combined group were decreased significantly compared with BLM (7.5 mg/kg) alone group (*p* < 0.05). These results suggested no significant difference in the levels of TNF-α and IL-6 between the model group and the SCU (60 mg/kg) alone group.

### Effects of SCU on the Levels of MPO and MDA

**FIGURE 8:**
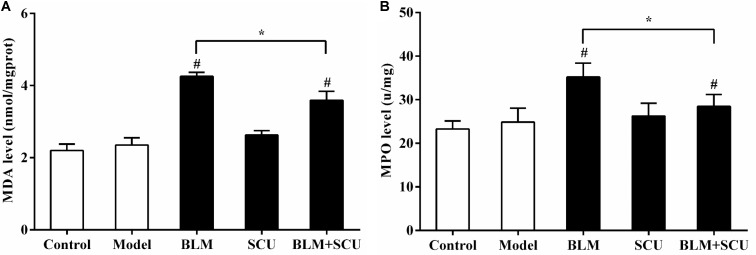
Effects of SCU, BLM, and their combination on the MPO **(A)**, MDA **(B)** levels in lung tissue of H22 tumor-bearing mice. Data are shown as the mean ± SD. (*n* = 8). ^#^*p* < 0.05 compared with model group; ^∗^*p* < 0.05 compared with BLM group.

To study whether SCU attenuation on BLM-induced pulmonary fibrosis was associated with antioxidant effects, the levels of MPO (**Figure 8A**) and MDA (**Figure 8B**) were assayed. In **Figure 8**, when compared with model group, the levels of MPO and MDA in BLM (7.5 mg/kg) alone group were significantly increased (*p* < 0.05). However, compared with BLM alone group, the levels of MPO and MDA in BLM (7.5 mg/kg) and SCU (60 mg/kg) combined group were remarkably decreased (*p* < 0.05). The above results suggested that the protective effects of SCU against BLM-induced lung injury may be related to antioxidant activity.

### Effect of BLM, SCU, and Their Combination on Immunofluorescence of α-SMA in MRC-5 Cells

**FIGURE 9:**
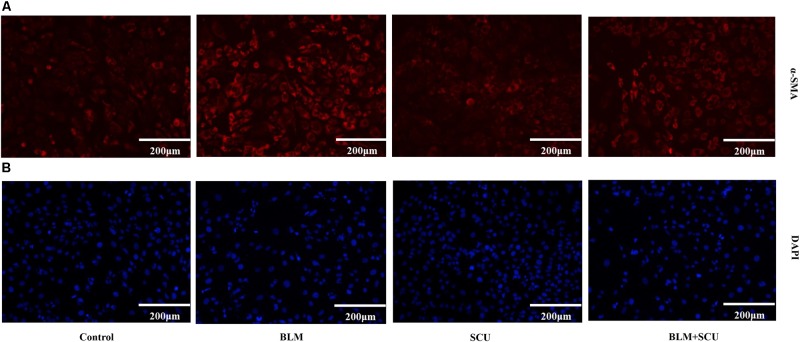
Effects of BLM, SCU, and their combination on the expression of α-SMA. (**A**, ×200) Immunofluorescence staining was performed to measure α-SMA protein expression (red) in MRC-5 cells. (**B**, ×200) The nuclei were stained by 4′,6-diamidino-2-phenylindole (DAPI) (blue). Representative images of each group are shown. Scale bar indicates 200 μm.

α-SMA is considered to be a marker of cell fibrosis. So, we used immunofluorescence to analyze the expression of α-SMA in MRC-5 cells, and the results are shown presented in **Figure 9**. When compared with control group, the expression of α-SMA in BLM group was significantly up-regulated. However, the expression of α-SMA in BLM combined with SCU group was significantly lower than the BLM alone group. A decrease in the expression of α-SMA indicated a decrease in the degree of pulmonary fibrosis. So, these results confirmed that SCU could reduce pulmonary fibrosis induced by BLM.

### Effect of SCU and BLM Treatments on p53, TGF-β1, α-SMA, and Collagen-I Expressions

**FIGURE 10:**
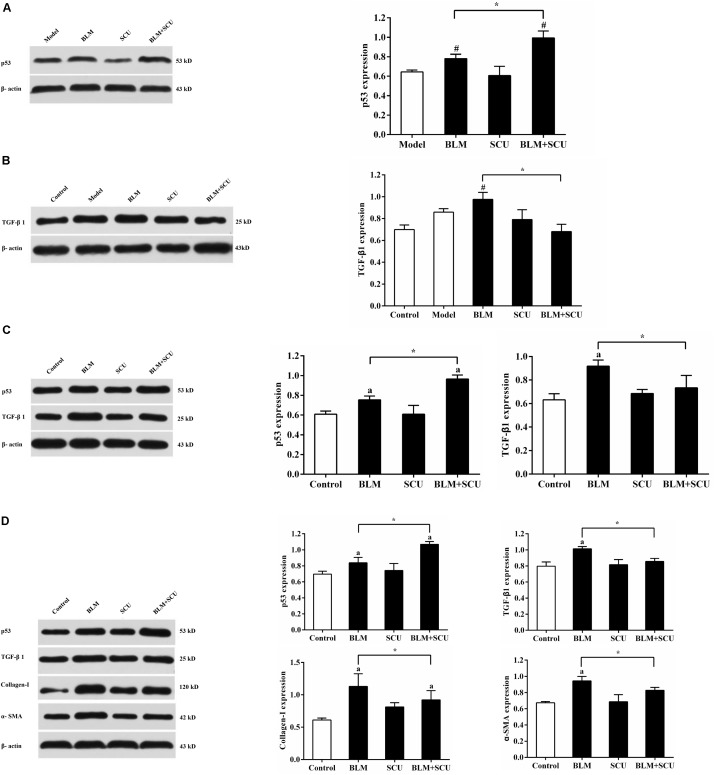
Effects of BLM, SCU, and their combination on the expression p53, TGF-β1 collagen-I and α-SMA. The representative images about protein expressions of p53, TGF-β1, collagen-I, α-SMA, and β-actin were measured by Western blot. **(A)** The expression of p53 in ascites. **(B)** The expression of TGF-β1 in lung tissues. **(C)** The expression of p53 and TGF-β1 in H22 cells. **(D)** The expression of p53, TGF-β1, collagen-I, and α-SMA in MRC-5 cells. Data are shown as the mean ± SD. (*n* = 3). ^#^*p* < 0.05 compared with model group; ^∗^*p* < 0.05 compared with BLM group; *a* < 0.05 compared with control group.

Results of protein expression of p53 and TGF-β1 in animal experiment are shown in **Figures 10A,B**. Compared with BLM alone group, the protein expression of p53 in the BLM combined with SCU group was increased and the protein expression of TGF-β1 in the BLM combined with SCU group was decreased (both *p* < 0.05). As shown in **Figure 10C**, when compared with BLM alone group, the SCU and BLM combined group significantly inhibited TGF-β1 expression in H22 cells and induced the expression of p53 in H22 cells (both *p* < 0.05). Furthermore, in **Figure 10D**, compared with control group, the expression levels of TGF-β1, p53, α-SMA, collagen-I in the BLM group were increased significantly (both *p* < 0.05) in MRC-5 cells. And the expression of TGF-β1, α-SMA, collagen-I in BLM combined with SCU group were significantly lower than that of the BLM alone group (both *p* < 0.05). However, the expression of p53 in BLM combined with SCU group was significantly increased, compared with BLM alone group (*p* < 0.05). The total un-cropped gels were provided in the Supplementary Material 2.

### Expression of miR-29b

**FIGURE 11:**
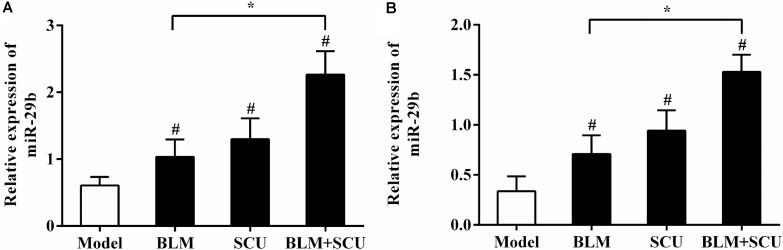
Effects of BLM, SCU, and BLM + SCU on the miR-29b levels in the lung tissues **(A)** and ascetic cells **(B)**. The miR-29b levels was detected by real-time quantitative PCR. Data are shown as the mean ± SD. (*n* = 4). ^#^*p* < 0.05 compared with model group; ^∗^*p* < 0.05 compared with BLM group.

Many studies have showed that miR-29b was closely associated with the development of tumors and pulmonary fibrosis (Cushing et al., 2015; Yan et al., 2015). So, our study attempted to investigate whether SCU could adjust to the miR-29b expression of BLM (7.5 mg/kg) alone group. As shown in **Figure 11**, when compared with the model group, the BLM and SCU (60 mg/kg) alone groups showed no obvious effects on the relative expression of miR-29b (*p* < 0.05). While the relative expression of miR-29b was significantly increased in the SCU (60 mg/kg) and BLM (7.5 mg/kg) combined group compared with BLM alone group (*p* < 0.05).

## Discussion

Nowadays, the incidence of cancer is increasing in a higher rate, and the number of cases in China is remains quite large. Tumors grow rapidly with infiltration and destruction of the structure and function of the surrounding tissues and in turn metastasizes, making it difficult to treat. Currently, chemotherapeutic drug therapy remains currently one of the main treatment strategy for cancer, but most of the chemotherapeutic drugs used clinically have varying degrees of immunosuppressive and bone marrow suppression side effects. Fortunately, as a broad-spectrum anti-cancer drug, BLM showed no bone marrow toxicity and immunosuppressive side effects (Sugiyama and Kumagai, 2002). However, a large number of clinical cases and experimental studies have shown that BLM was associated with lung toxicity (Froudarakis et al., 2013). Therefore, to safely use BLM clinically, it is necessary to find a drug or adjuvant that can reduce the side effects of BLM.

In recent years, more and more researchers began to seek cancer drugs from nature medicine, as these are associated with low toxic effects. There are more than 4,000 flavonoids found till date, and are widely found in plants and berries. Studies showed that flavonoids have anti-tumor properties, anti-atherosclerotic effects, anti-inflammatory effects, anti-thrombogenic effects, antiviral effects and antibacterial effects (Sankari et al., 2014). Research data showed that flavonoids combined with anti-cancer drugs produced synergistic as well as attenuated effects (Yang et al., 2015). For example, combination of doxorubicin and quercetin copolymer has synergistic antitumor effect and reduces cardiotoxicity (Qureshi et al., 2016). SCU, commonly used for the treatment of cerebrovascular diseases, is a flavonoid extracted from *Erigeron breviscapus* (Vant.) Hand-Mazz. Modern pharmacological experiments showed that SCU have anti-inflammatory properties, suppressing the growth, migration, and invasion of cancer cells (Li et al., 2010; Yuan et al., 2014; Ke et al., 2017). So, we hypothesized that SCU can increase the anti-tumor effect of BLM, while alleviating the lung toxicity of BLM. Our experimental data indicated that BLM combined with SCU can significantly improve the weight and decrease the abdominal diameter and ascites volume of experimental mice, compared to model group. However, there was no significant antitumor effect when SCU was used alone. These results demonstrated that SCU could increase the anti-tumor effect of BLM.

Apoptosis is an indispensable process of life, which involves spontaneous death of cells, and has biological significance. As tumor cells have a strong ability to proliferate, metastasize, and invade, a potential strategy in the treatment of tumors is to induce apoptosis in cancer cells. The literature also shows that activation of apoptosis was associated with the development and treatment of tumors (Chresta and Hickman, 1999). Previous studies have revealed that SCU could induce apoptosis and suppress migration and invasion of human hepatocellular carcinoma by inhibiting the STAT3/Girdin/Akt signaling pathway (Xu and Zhang, 2013; Ke et al., 2017). In this study, to further clarify the mechanism of SCU improving the anti-tumor effects of BLM, the apoptotic rate of H22 ascites cells were detected through *in vitro* and *in vivo* experiments. Our experimental results showed that BLM combined with SCU increased the apoptotic rate of H22 cells. Caspase family is closely related to cell apoptosis. Caspase-3 is thought to be the most important terminal shearing enzyme in the process of apoptosis. Studies have shown that an increase in caspase-3 activation may indicate an increase in apoptosis (Hu et al., 2000). Caspase-8 has been shown to activate caspase-3 and other caspase family proteins (Wu et al., 2011). And the activated caspase-8 is known to direct activation or cleavage downstream caspase to transmit apoptotic signals, causing apoptosis (Kruidering and Evan, 2000). Previous studies have shown that SCU could induce apoptosis of colon cancer cells by enhancing caspase-6 activity (Chan et al., 2009). Our study results showed that BLM combined with SCU treatment significantly enhanced the levels of cleaved-caspase-3 and cleaved-caspase-8. Furthermore, as a crucial apoptotic protein, p53 can mediate the downstream cleaved-caspase-3 and cleaved-caspase-8 directly. And more recently, SCU has been confirmed to induce cancer cell apoptosis through p53 and other pathways (Feng et al., 2012). Here, we found that SCU combined with BLM could notably enhance the expression of p53 in H22 ascites cells and *in vitro* H22 cells. These results suggested that SCU combined with BLM induced apoptosis of H22 tumor cells might be by activation of the p53 apoptotic signaling pathway.

Bleomycin has broad spectrum anti-cancer effects, and produces no immunosuppression and bone marrow suppression. But BLM treatment induces pulmonary fibrosis, and remains to be the biggest problem. Pulmonary fibrosis is a serious lung disease that is characterized by fibroblast proliferation, patchy parenchymal inflammation, epithelial cell injury with reactive hyperplasia, basement membrane, and alveolar epithelial injury (Della Latta et al., 2015). Inflammation is an early symptom of pulmonary fibrosis, and inflammatory alveolar infiltration of inflammatory cells occurs during this process. In addition, inflammatory reactions produce different inflammatory factors and these directly reflect the degree of inflammatory response. TNF-α and IL-6 are common inflammatory cytokines (Hibi et al., 1996; Shohami et al., 1999; Straub et al., 2000). Della Latta et al. (2015) demonstrated that there was a significant up-regulation of TNF-α and IL-6 in BLM-induced pulmonary fibrosis. Our results showed that in the SCU combined with BLM group, the levels of TNF-α and IL-6 were distinctly decreased. Moreover, oxidative stress is also involved in the development of inflammation. MPO and MDA are typical enzymes and products of oxidative stress and play an important role in the inflammatory response. This study displayed that SCU combined with BLM could down the levels of MPO and MDA. Furthermore, as the inflammatory-immune response progresses, inflammation and abnormal repair lead to the proliferation of pulmonary interstitial cells, producing large amounts of collagen and extracellular matrix, such as α-SMA and collagen-I. Research studies have shown that pulmonary fibrosis in rats with fibrosis is accompanied by the overexpression of α-SMA and collagen-I (Cabalgante et al., 2012; Li et al., 2015). In order to reflect the degree of pulmonary fibrosis and whether SCU can treat pulmonary fibrosis, we measured the expression of α-SMA and collagen-I in MRC-5 cells cultured *in vitro*. Experimental results revealed that the expression of α-SMA and collagen-I in MRC-5 cells were decreased in the BLM combined with SCU group. Interestingly, previous data reported that TGF-β1, the growth-inhibitory cytokine, could suppress the release of TNF-α and IL-6. In turn, TNF-α and IL-6 can also affect the TGF-β1 activity (Zhao et al., 2010). And in a rat model of BLM-induced pulmonary fibrosis, TGF-β1 can regulate the level of MDA and MPO (Altintas et al., 2016). Furthermore, TGF-β1 has been reported to induce the expression of α-SMA and collagen-I in MRC-5 cells (Park et al., 2016). Moreover, SCU can alleviates interstitial fibrosis through inhibiting TGF-β1 expression (Pan et al., 2011) and prevents Diosbulbin B induced liver injury by attenuating NF-κB mediated hepatic inflammation and ameliorating liver oxidative stress injury (Pan et al., 2011; Niu et al., 2015). In the present study, the expression of TGF-β1 in lung tissue and MRC-5 cells in the SCU combined with BLM group was evidently decreased. These results showed that SCU could alleviate the side effects of BLM by regulating the TGF-β1 signaling pathway.

TGF-β1 signaling pathway is well known for its ability to control a variety of cellular processes, including cell recognition, proliferation, differentiation and apoptosis (Dong and Blobe, 2006). The incidence of cancer mostly occurs due to malignant proliferation and metastasis of cells. Therefore, many researchers believe that the development and treatment of cancer initiates from the induction of cancer cell apoptosis. Now that, a lot of literature has proved TGF-β1 signaling pathway in the induction of cancer through multiple expression of apoptotic, autophagy, and proliferating genes (Markowitz and Roberts, 1996; Roberts and Wakefield, 2003; Seoane and Gomis, 2017). Also, TGF-β1 can affect the activities of MDA, MPO, and TGF-β1 and associated inflammatory factors, such as TNF-α and IL-6 (Zhao et al., 2010; Altintas et al., 2016). In addition, TGF-β1 signaling is associated with pulmonary fibrosis (Gordon and Blobe, 2008). Furthermore, another important tumor-related signaling pathway is the p53 signaling pathway. P53 is a tumor suppressor gene, and is related to the occurrence or loss of mutations in more than 50% of the cancer types. Studies have shown that p53 can also be involved in the cell cycle and senescence processes (Hock and Vousden, 2012). P53 have revealed that can regulate cell cycle arrest and promote cell differentiation, apoptosis, and DNA repair (Muller et al., 2011; Gurpinar and Vousden, 2015). Moreover other experimental data showed that p53 signaling pathway can treat cancer by inducing apoptosis of cancer cells, preventing AEC damage caused by silica-induced lung injury (Vousden, 2002; Bhandary et al., 2015). P53 was also associated with BLM-induced pulmonary fibrosis (Ghosh et al., 2002) and has been shown to suppress pulmonary fibrosis by activating p53 gene in the induction of apoptosis in lung fibroblasts (Zhang et al., 2015). In addition, several literature reports indicated that p53 and TGF-β1 were potentially associated with cell apoptosis and tumor genesis (Cordenonsi et al., 2003; Wilkinson et al., 2005). The previous data have suggested that p53 signaling pathway interacts with TGF-β1 signaling pathway in cancer and pulmonary fibrosis (Elston and Inman, 2012). So combined our experimental results, we hypothesized that SCU through TGF-β1 and p53 pathway enhances the anti-tumor efficacy of BLM and reduces the toxic effects of BLM.

Recent study has exposed a key role of miR-29b in tumor cell apoptosis, differentiation, migration in different types of cancers (Qi and Huang, 2017). And miR-29b was reported to associate with the BLM-induced pulmonary fibrosis (Montgomery et al., 2014) and associated with the occurrence of pulmonary fibrosis by adjusting the expression of collagen, matrix metallopeptidase, inflammatory cytokines and participating in TGF-β1 pathway (Cushing et al., 2015). TGF-β1 signaling pathway in turn affects the occurrence of pulmonary fibrosis by the expression of miR-29b. In addition, studies have shown that miR-29b induces the apoptosis of tumor cells by activating downstream the p53 pathway including caspase-3 and -8 (Rebbaa et al., 2001; Mathe et al., 2012). Also, miR-29b can participate in TGF-β1 pathway and p53 pathway in different ways (Park et al., 2009; Sivadas and Kannan, 2014). So, we hypothesized that miR-29b may play a key junction between p53 signaling and TGF-β1 signaling in cancer and pulmonary fibrosis. We measured the expression of miR-29b gene in lung tissues and ascites cells. Results demonstrated that the expression level of miR-29b gene in lung tissues and ascites cells was significantly increased when SCU was administered in combination with BLM. This indicated that SCU combined with BLM affected the expression level of miR-29b and regulated the balance between p53 and TGF-1 signaling pathways in H22 tumor-bearing mice.

Finally, considering the results obtained in this study, we hypothesis that SCU can significantly improve the anti-tumor effects of BLM and reduce the side effects of BLM when combined with SCU. The specific mechanism of SCU involves regulation of the balance of TGF-β1 and p53 signaling pathways, which in turn affects the expression of miR-29b in the induction of tumor cell apoptosis and inhibit the occurrence of pulmonary fibrosis.

There are still some limitations in our study. In the present study, we used the H22 ascites tumor model, H22 and MRC-5 cell models to explore the synergistic anti-tumor activity and attenuated effect of SCU in combination with BLM, and the possible mechanisms. However, we did not test the CI in more several cancer cell lines to confirm the synergistic anti-tumor activity. We did not confirm the attenuated effect of SCU in combination with BLM on the pulmonary fibrosis using more several cell lines (such as immune cells) and experimental animal models. In addition, we studied that SCU enhanced the anti-tumor efficacy of BLM and reduced the side effects of BLM through regulating TGF-β1 and p53 pathways. However, we have not studied in more detail about how SCU and BLM affect TGF-β1 and p53 signaling. Furthermore, in this study we only measured the expression of miR-29b and did not detect the expression of other miRNAs. Therefore, it is unclear whether combination of BLM and SCU affects the expression of other miRNAs. So further research regarding these issues is needed.

## Conclusion

Our present experimental results confirmed that SCU can enhance the anti-tumor effects of BLM and alleviate the toxic adverse effects of BLM when combined with SCU for the treatment of tumor. The possible mechanism of SCU may occur through the regulation of balance of p53 and TGF-β pathways to achieve the purpose of increasing the efficiency and reducing the toxicity. These indicate that SCU can be used as an adjuvant in combination with chemotherapeutic agents for the treatment of cancers.

## Author Contributions

Y-CL and Z-RS conceived and designed the experiments. JN, H-MY, C-YS, and Z-BZ performed the experiments. Y-LL and J-YZ analyzed the data. Y-CL, Z-RS, and X-PL were the group leader offering supervision and financial support. JN and Y-CL wrote the paper. JN, H-MY, and Y-CL supplemented the experiments and revised the paper. All authors reviewed the manuscript.

## Conflict of Interest Statement

The authors declare that the research was conducted in the absence of any commercial or financial relationships that could be construed as a potential conflict of interest. The handling Editor and reviewer AT declared their involvement as co-editors in the Research Topic, and confirm the absence of any other collaboration.
