# Supplementary material for: Scutellarin Enhances Antitumor Effects and Attenuates the Toxicity of Bleomycin in H22 Ascites Tumor-Bearing Mice
Source: Front Pharmacol. 2018 Jun 14;9:615. doi: 10.3389/fphar.2018.00615 (PMC6011816; doi:10.3389/fphar.2018.00615)
Supplement: Supplementary file 1 [file Data_Sheet_1.docx]

**Supplementary material 1**

**Scutellarin enhances antitumor effects and attenuates the toxicity of Bleomycin in H22 ascites tumor-bearing mice**

**Juan Nie ^1, #^, Hong-Mei Yang ^1, #^, Chao-Yue Sun ^2^, Yan-Lu Liu ^1^, Jian-Yi Zhuo ^1^, Zhen-Biao Zhang ^1^, Xiaoping Lai ^1, 3^, Zi-Ren Su ^1, 3, *^, Yu-cui Li ^1, 3, *^**

^1^ Mathematical Engineering Academy of Chinese Medicine, Guangzhou University of Chinese Medicine, Guangzhou, 510006, China

^2^ Guangdong Province Traditional Chinese Medical Hospital, Guangzhou, 510006, China

^3^ Guangdong Provincial Key Laboratory of New Drug Development and Research of Chinese Medicine, Guangzhou University of Chinese Medicine, Guangzhou 510006, China;

^#^ These authors contributed equally to this work

^*^ These corresponding authors contributed equally to this work

**Correspondence to:** Zi-Ren Su

[suziren@gzucm.edu.cn](mailto:suziren@gzucm.edu.cn)

**Correspondence to:** Yu-Cui Li

[liyucui@gzucm.edu.cn](mailto:liyucui@gzucm.edu.cn)

**Preliminary experiment for** **the BLM administration time**

H22 cells (2×10^6^ cells/ml) were inoculated into the abdomen of male KM mice and the ascites cells were passaged three times in the mice, after one week, the ascites was collected and diluted with normal saline; the cell concentration was adjusted to 2×10^6^ cells/ml and injected into each mouse. After 5 days, the 30 mice were randomly divided into 3 groups with 10 mice in each group: the control group (intraperitoneal (ip) injection of normal saline), model group (normal saline, ip), BLM alone group (7.5mg/kg, ip). At day 8, 10 mice of each group were executed and their lungs were rapidly removed and cleaned. Subsequently, lungs tissues were photographed. Then lung tissues were fixed in 10% neutral buffered formalin and embedded in paraffin wax, cut into 5 μm thick slices, and subjected to haematoxylin-eosin (H&E) staining and Masson’s trichrome staining to detect inflammation or collagen deposition, respectively.

As shown in Suppl. Figure S1, in the photographs of the lung tissue, we can see that the control group (A) didn’t show appearance of pulmonary edema or fibrosis in the mice. However, the BLM group (B) had obvious fibrosis and edema in the lung tissues. In addition, H&E staining **(**C**)** presented obvious pulmonary injury, including alveolar wall, alveolar, vascular, congestion, and inflammatory cell infiltration in the BLM alone group. Moreover, Masson’s trichrome staining (D) suggested that BLM alone group had massive collagen deposition in the lung interstitium and around the bronchioles as compared to the model group. Therefore, a period of 7 days’ administration of BLM was selected for this study.

**Preliminary experiment for the BLM and SCU dose selection in vitro**

[3-(4,5-dime-thylthiazol-zyl)-5-(3-carboxymethoxyphenyl)-2-(4-sulfophenyl)-2H-tetrazoli-uzolium, inner salt] (MTS) (Sigma-Aldrich Co) assay was used to measure the inhibition rate of SCU, BLM in H22 cells. H22 cells (1×10^5^ cells/ml) were plated in 96-well plates with 200 µl in each well. RPMI 1640 medium was added into the blank group without cells, the control group with only cells, and the experimental group containing BLM (1.25 µM, 2.5 µM, 5 µM, 10 µM, 20 µM), SCU (1.25 µM, 2.5 µM, 5 µM, 10 µM, 20 µM). After culturing for 24 h and 48 h, 20 µl of MTS was added to each well. The culture was then incubated continuously for 4 h, and the optical density (OD) was measured with micro-plate reader at a wavelength of 490 nm.

As shown in Suppl. Figure S2, we can see when the dose of BLM was less than 5 µM, BLM didn’t show significant inhibitory effect on H22 cells, and SCU also showed the same result. Therefore, we eventually dosed from 5 µM.

**Preliminary experiment for the BLM dose selection**

H22 cells (2×10^6^ cells/ml) were inoculated into the abdomen of male KM mice and the ascites cells were passaged three times in the mice, after one week, the ascites was collected and diluted with normal saline; the cell concentration was adjusted to 2×10^6^ cells/ml and injected into each mouse. After 5 days, the 50 mice were randomly divided into 5 groups with 10 mice in each group: the control group (intraperitoneal (ip) injection of normal saline), model group (normal saline, ip), BLM - L, M, H doses alone group (5, 7.5, 15 mg/kg, respectively, ip). All the mice were allowed free access to water and food until death, and the survival rate was calculated.

As shown in Suppl. Figure S3, BLM-H alone group exhibited no significant influence on the life-span of the tumor-bearing mice when compared with BLM-M alone group. However, the BLM-M alone group could significantly prolong the survival time of mice as compared to control group and BLM-L alone group (*p* < 0.05). Therefore, 7.5 mg/kg of BLM were used in this study.

**Figure Legends**

**Suppl. Figure S1. Mouse Lung tissue changes after treatment with BLM (7.5mg/kg) and normal saline for 7 days.** **(A)** shows lung tissue changes of three mice in control group (treated with normal saline). **(B)** shows lung tissue changes of three mice in BLM (7.5mg/kg) alone group. Lung tissue sections were stained with haematoxylin-eosin (H&E) **(C)** for pathological examination (200×); Masson **(D)** for collagen deposition (200×); Scale bar indicates 50 μm.

**Suppl. Figure S2. Preliminary experiment for the BLM and SCU dose selection in vitro. (A)** The inhibitory rate of BLM on H22 cells after 24 h. **(B)** The inhibitory rate of SCU on H22 cells after 24 h. The inhibitory rate =1- (OD _Experimental_-OD _Blank_/OD _Control_-OD _Blank_ × 100%). n=5.

**Suppl. Figure S3. Preliminary experiment for the BLM administration selection.** The survival rate followed-up until 22 days after inoculation. Each group comprised of eight mice. Log-rank (Mantel-Cox) test was applied to analyze the comparison of survival curves. n=8. * *p* < 0.05 compared with control group; ^#^ *p* < 0.05 compared with BLM-L alone group.

**Suppl. Figure S1.**


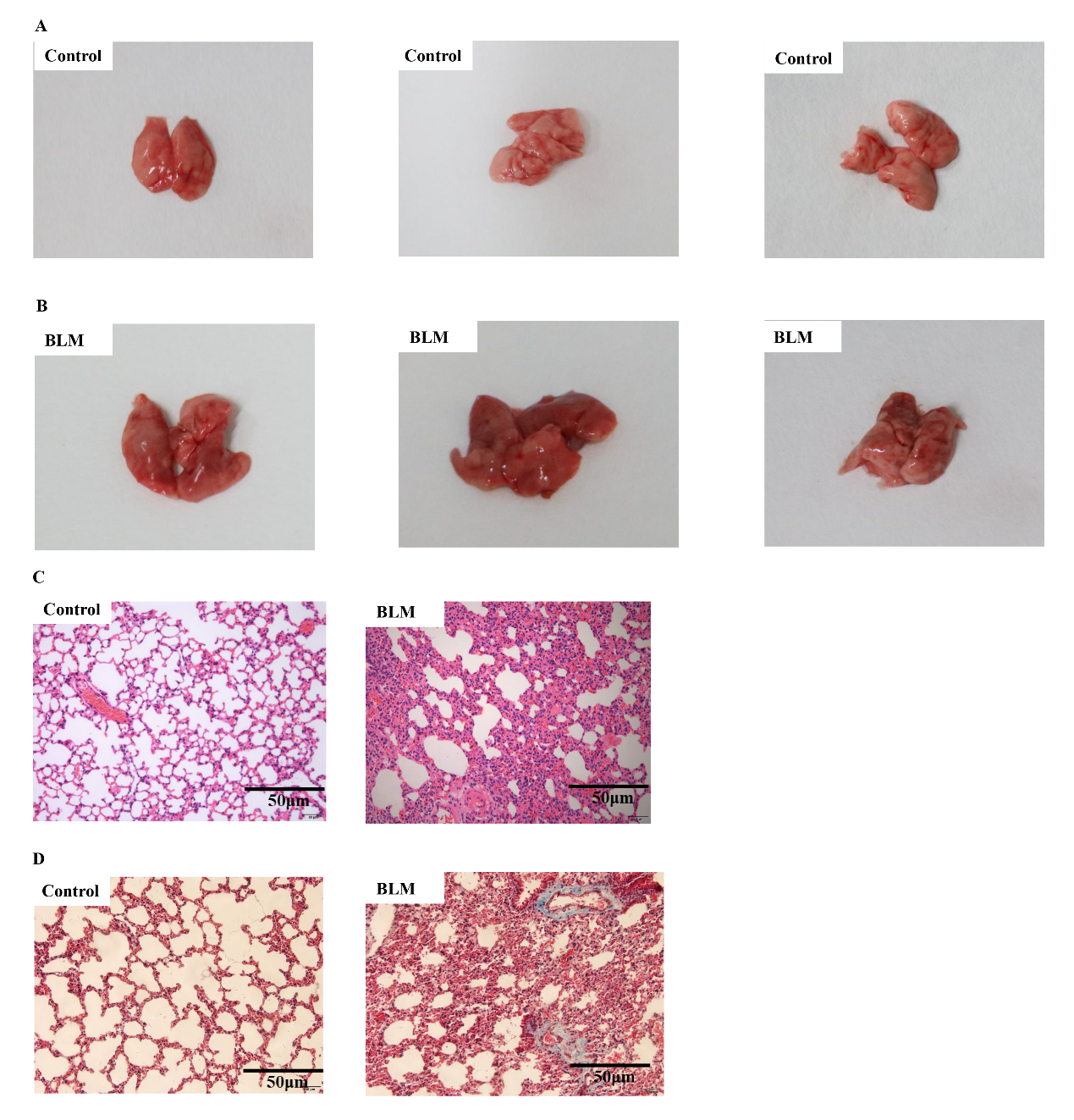


**Suppl. Figure S2.**


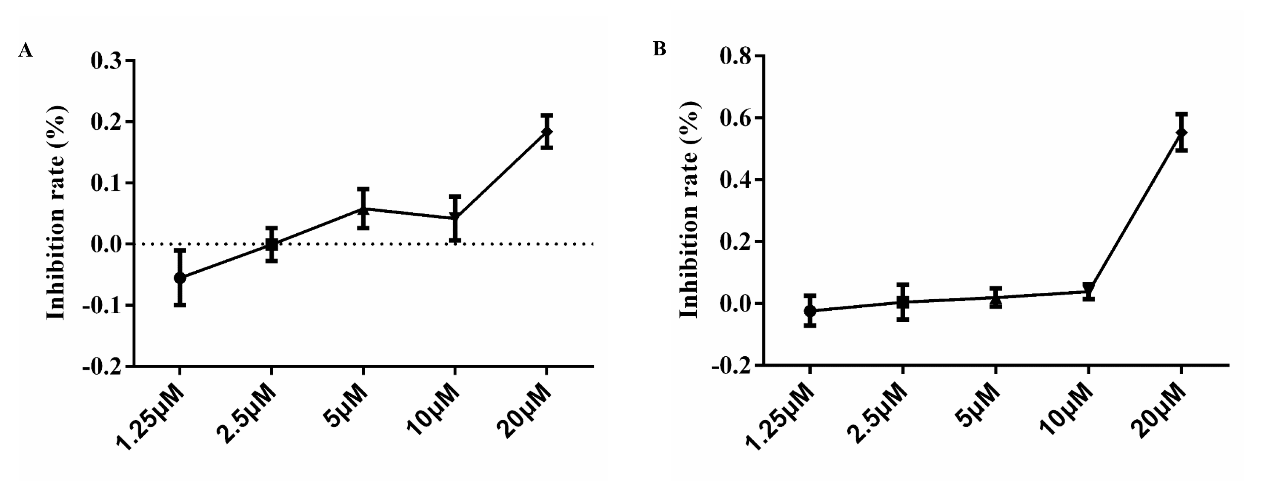


**Suppl. Figure S3.**
