# Supplementary material for: Scutellarin Enhances Antitumor Effects and Attenuates the Toxicity of Bleomycin in H22 Ascites Tumor-Bearing Mice
Source: Front Pharmacol. 2018 Jun 14;9:615. doi: 10.3389/fphar.2018.00615 (PMC6011816; doi:10.3389/fphar.2018.00615)
Supplement: Supplementary file 2 [file Data_Sheet_2.docx]

**Supplementary material 2**

**Scutellarin enhances antitumor effects and attenuates the toxicity of Bleomycin in H22 ascites tumor-bearing mice**

**Juan Nie ^1, #^, Hong-Mei Yang ^1, #^, Chao-Yue Sun ^2^, Yan-Lu Liu ^1^, Jian-Yi Zhuo ^1^, Zhen-Biao Zhang ^1^, Xiaoping Lai ^1, 3^, Zi-Ren Su ^1, 3, *^, Yu-cui Li ^1, 3, *^**

^1^ Mathematical Engineering Academy of Chinese Medicine, Guangzhou University of Chinese Medicine, Guangzhou, 510006, China

^2^ Guangdong Province Traditional Chinese Medical Hospital, Guangzhou, 510006, China

^3^ Guangdong Provincial Key Laboratory of New Drug Development and Research of Chinese Medicine, Guangzhou University of Chinese Medicine, Guangzhou 510006, China;

^#^ These authors contributed equally to this work

^*^ These corresponding authors contributed equally to this work

**Correspondence to:** Zi-Ren Su

[suziren@gzucm.edu.cn](mailto:suziren@gzucm.edu.cn)

**Correspondence to:** Yu-Cui Li

[liyucui@gzucm.edu.cn](mailto:liyucui@gzucm.edu.cn)

**Figure legends**

**Supp2. Figure S1. The total un-cropped gels. (A)** The expression of p53 in H22 ascites cells. **(B)** The expression of TGF-β1 in lung tissues. **(C)** The expression of p53 and TGF-β1 in H22 cells cultured in vitro. **(D)** The expression of p53 and α-SMA in the MRC-5 cells cultured in vitro. **(E)**The expression of Collgen-Ⅰ and TGF-β1 in MRC-5 cells cultured in vitro.

**Supp2. Figure S1.**

**
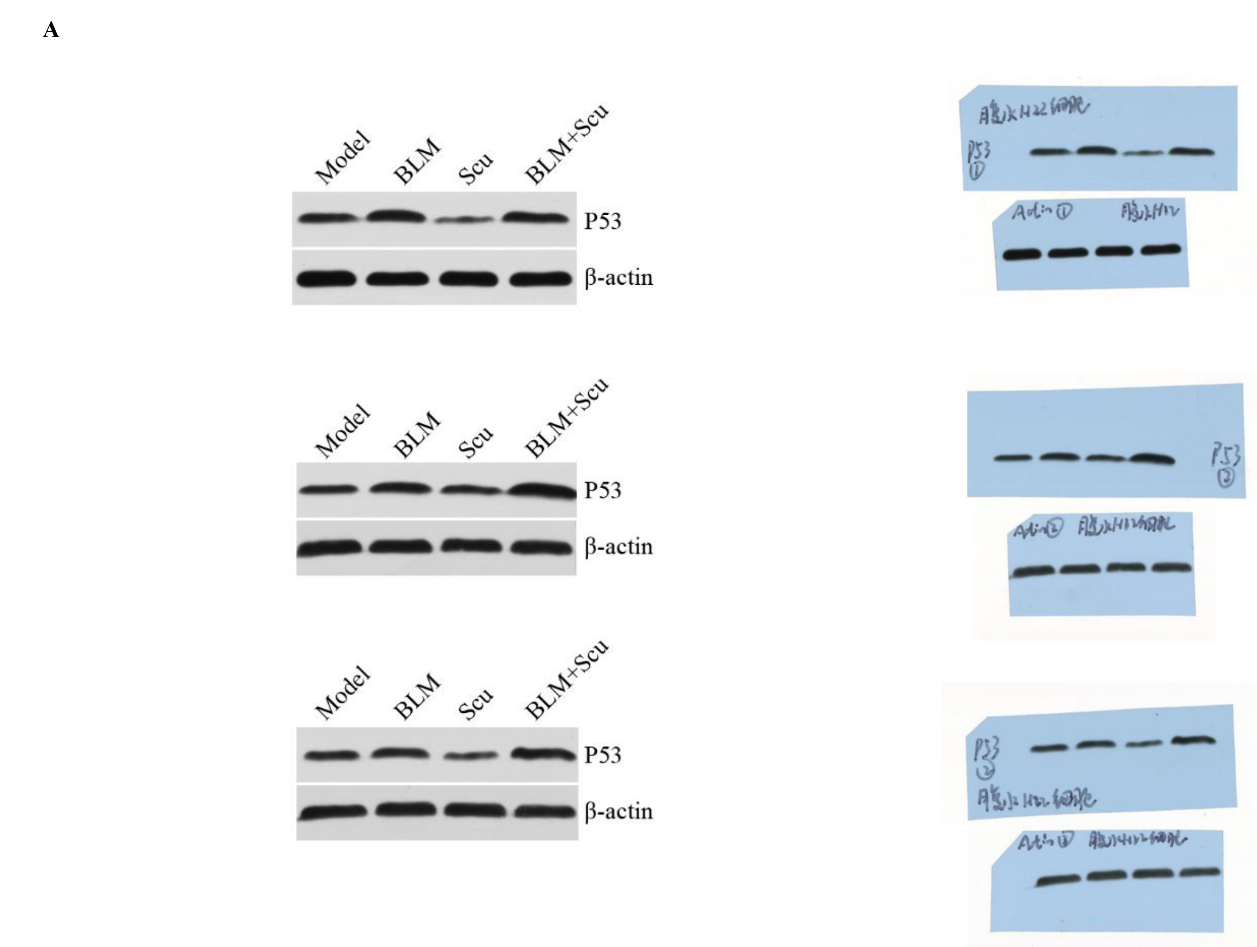
**

**
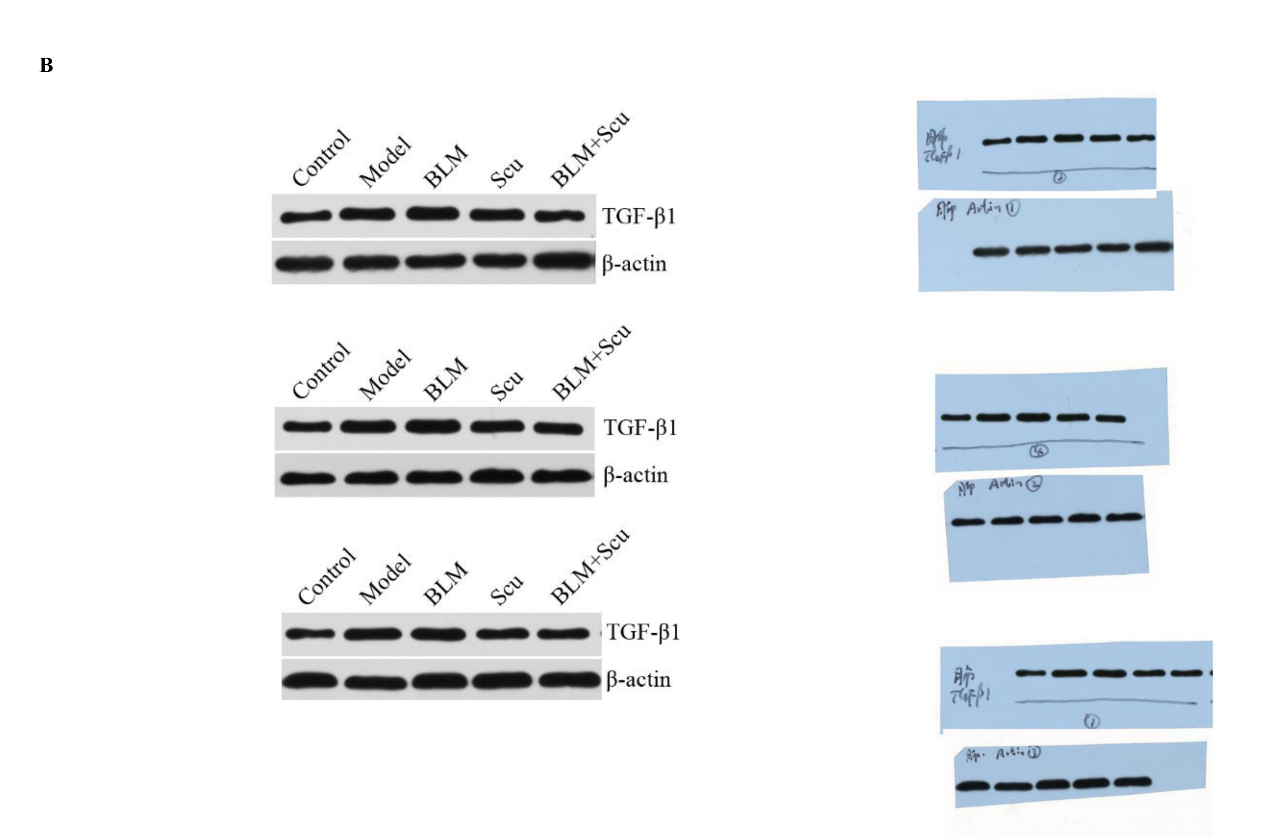
**

**
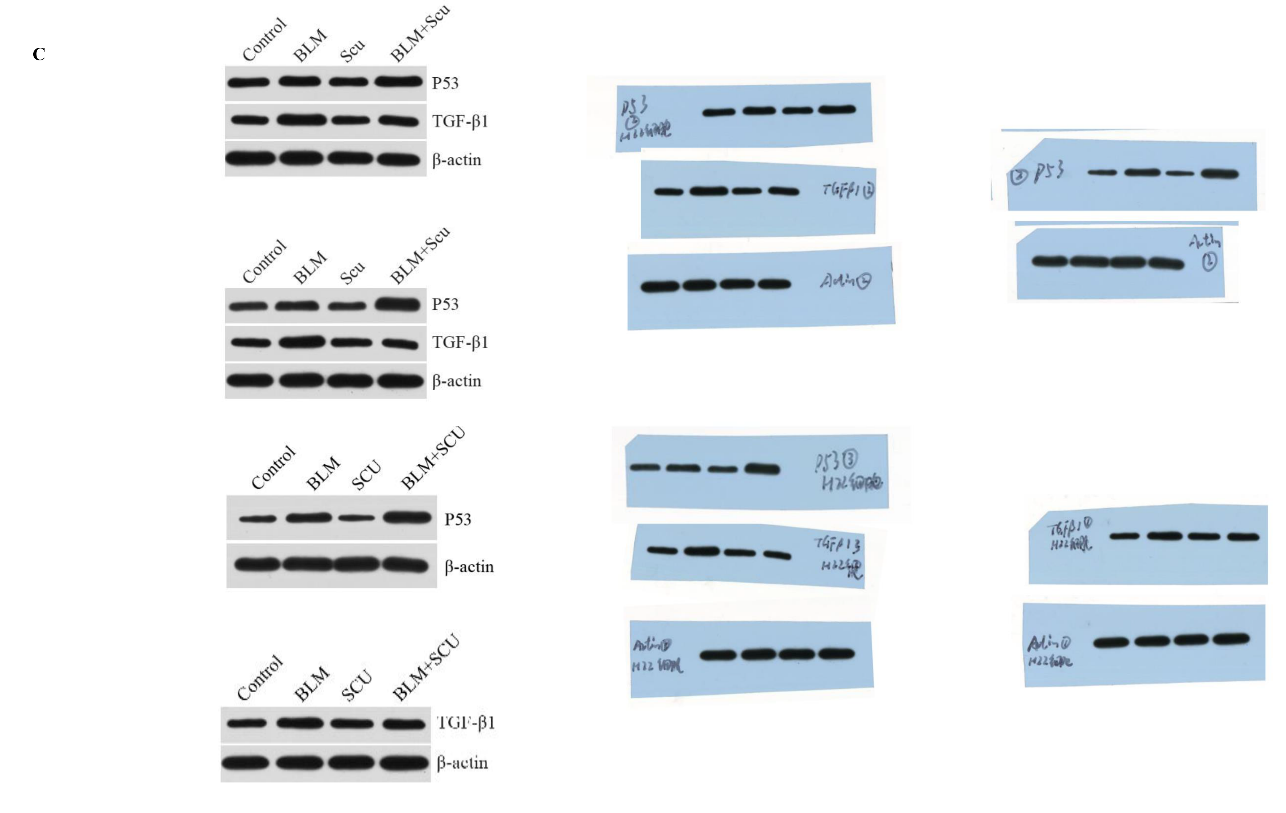
**

**
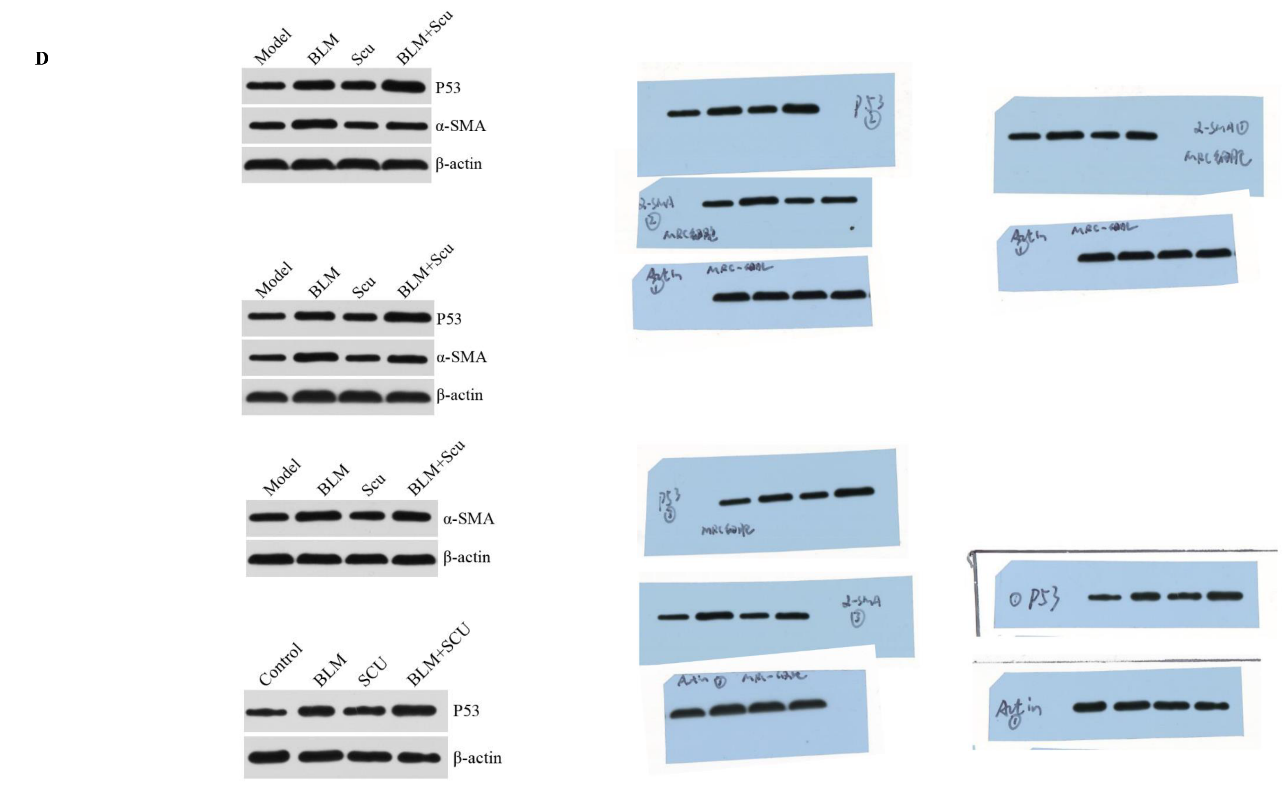
**

**
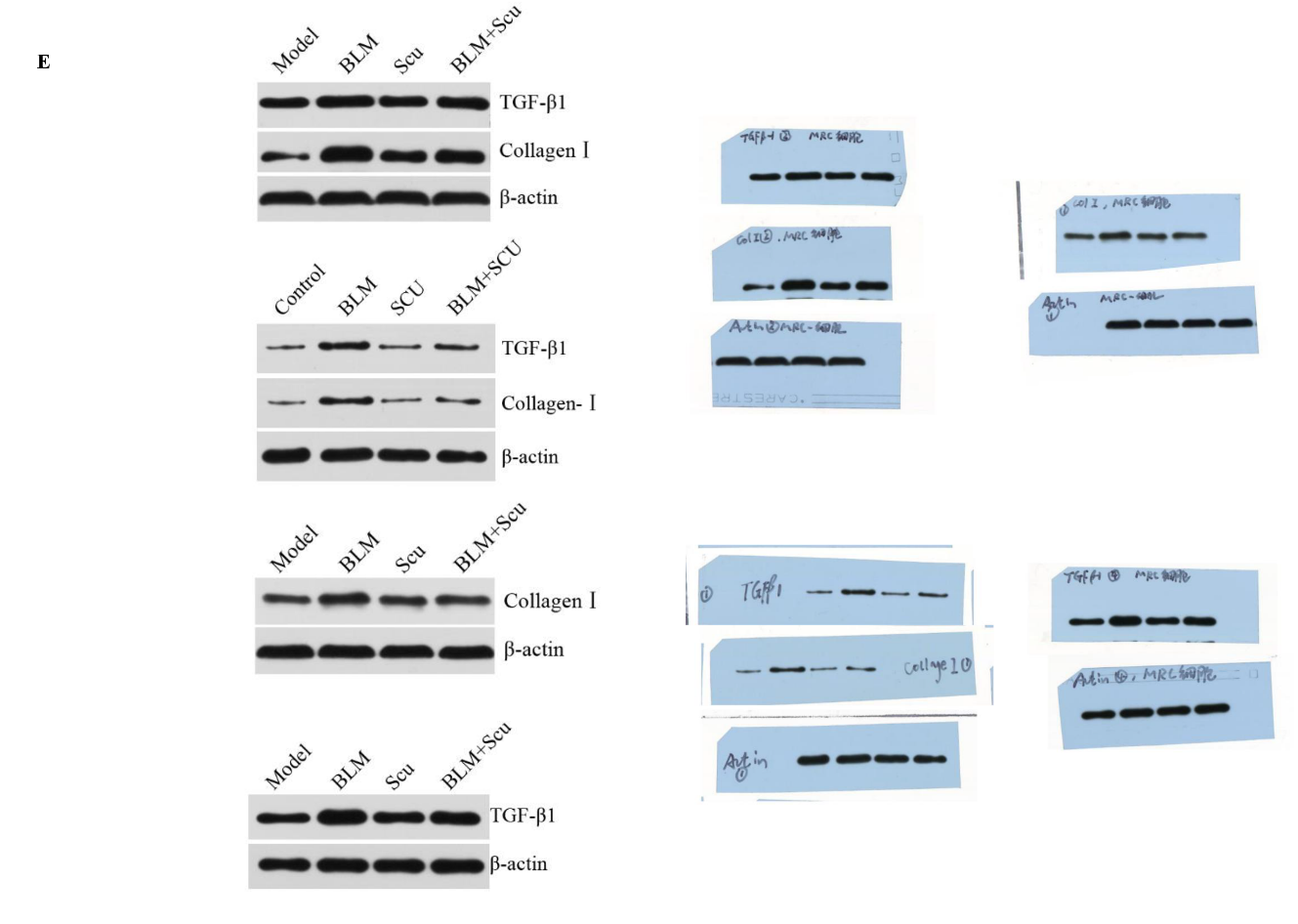
**
